# Crosstalk between acetylation modification and autophagy in cancer: roles, mechanisms, and therapeutic potential

**DOI:** 10.1038/s41420-025-02809-x

**Published:** 2025-11-10

**Authors:** Yingnan Liu, Ziwei Yan, Zhanqi Fu, Xiaoyang Wu, Fang Wang, Yuan Yuan

**Affiliations:** 1 Tumor Etiology and Screening Department of Cancer Institute and General Surgery, The First Hospital of China Medical University, Shenyang, Liaoning China; 2 Key Laboratory of Cancer Etiology and Prevention in Liaoning Education Department, The First Hospital of China Medical University, Shenyang, China; 3 Key Laboratory of GI Cancer Etiology and Prevention in Liaoning Province, The First Hospital of China Medical University, Shenyang, China; 4 Department of Pathology, Qilu Hospital of Shandong University Dezhou Hospital, Dezhou, Shandong China

**Keywords:** Targeted therapies, Autophagy, Drug development

## Abstract

Acetylation modification and autophagy are fundamental mechanisms regulating cell fate and homeostasis, exhibiting a highly coordinated and dynamic interplay in cancer development. Emerging studies have revealed that acetylation modulates the activation and inhibition of autophagy by regulating the activity, stability, and subcellular localization of autophagy-related proteins. Conversely, autophagy reciprocally influences cellular acetylation levels through selective degradation of acetyltransferases and deacetylases, as well as modulation of acetyl-CoA metabolism, forming a complex bidirectional regulatory network. In cancer, this crosstalk critically contributes to metabolic reprogramming, migration and invasion, therapeutic resistance, and adaptation to the tumor microenvironment, thereby influencing tumor progression and prognosis. This review systematically summarizes the functional roles and interaction mechanisms of acetylation and autophagy across various cancer types, with a particular focus on small-molecule agents targeting this axis and their current status in clinical applications. Although these therapeutic strategies have demonstrated promising anti-tumor potential in both preclinical and clinical settings, challenges such as limited drug specificity, mechanistic heterogeneity, and acquired resistance remain to be addressed. Future research should explore non-canonical forms of acetylation, immune regulation within the tumor microenvironment, and personalized therapeutic models, aiming to identify key regulatory nodes in the acetylation–autophagy network and unlock their potential for precision cancer therapy.

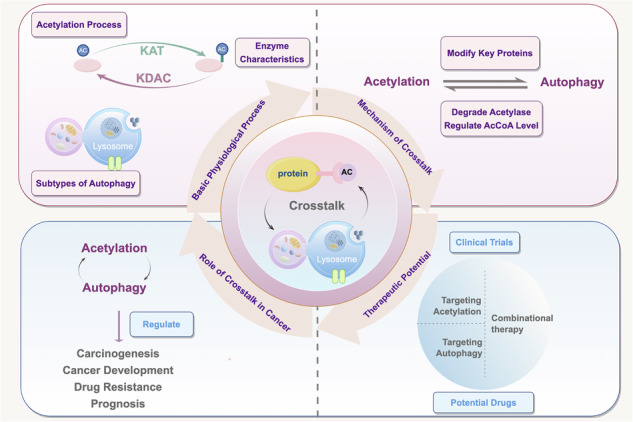

## Facts

- Acetylation and autophagy are two critical regulatory processes involved in cellular homeostasis, stress responses, and disease pathogenesis. Their crosstalk serves as a fundamental adaptive mechanism that enables cells to maintain homeostasis and respond to environmental changes, playing an essential role in cancer development and progression.
- In cancer, the intricate bidirectional crosstalk between acetylation and autophagy plays a pivotal role in tumor cell survival, metastasis, and drug resistance through mechanisms such as metabolic reprogramming, DNA repair modulation, and immune microenvironment remodeling.
- Acetylation dynamically regulates autophagy initiation and termination by modulating the activity and stability of autophagy-related proteins, while autophagy, in turn, influences acetylation homeostasis by promoting the degradation of acetyltransferases/deacetylases or altering metabolic substrate availability.
- Therapeutic strategies targeting both acetylation and autophagy have shown promising anti-cancer potential in preclinical models and clinical trials. Research in this field is now at a critical juncture, transitioning from fundamental mechanistic studies to clinical translation, with the goal of advancing precision oncology.

## Open Questions

- How does non-classical acetylation modification (e.g., N-terminal acetylation) influence autophagosome formation and substrate selection?
- What are the cooperative mechanisms between acetylation and autophagy in organelle-specific autophagy, such as mitophagy and ER-phagy?
- How can the heterogeneity of autophagy (e.g., selective vs. non-selective autophagy) and transient acetylation events within microcompartments (e.g., lysosomal membranes, autophagosomes) be captured?
- What strategies can be employed to develop subtype-selective modulators of acetyltransferases and deacetylases, minimizing off-target effects and overcoming tumor adaptive resistance?
- How does the acetylation-autophagy axis regulate immune cell function, and how can it be leveraged to enhance anti-tumor immune responses and effectively reverse the immunosuppressive tumor microenvironment?

## Introduction

In cell biology, the interplay between distinct molecular mechanisms, termed “crosstalk,” is fundamental for cellular homeostasis and adaptation to environmental changes [1]. Acetylation and autophagy are two key regulatory processes involved in cellular homeostasis, stress responses, and disease pathogenesis [2, 3]. Acetylation regulates protein activity, stability, subcellular localization, and molecular interactions, thereby influencing gene expression, metabolic regulation, and signal transduction [4]. Autophagy, on the other hand, degrades damaged or superfluous cellular components to enable recycling and clearance [5].

Recent studies have uncovered a complex bidirectional relationship between acetylation and autophagy: acetylation directly regulates the function and stability of key autophagy-related proteins, modulating autophagy initiation and progression [5, 6], while autophagy reciprocally regulates acetylation dynamics by degrading aberrantly acetylated proteins or modulating deacetylase activity [7–9]. This article comprehensively explores the physiological roles and mechanisms of acetylation and autophagy, analyzes their crosstalk in cancer, and reviews the clinical applications of drugs targeting this axis to evaluate its therapeutic potential.

## Overview of acetylation and autophagy

### Concept and functions of acetylation modification

**Fig. 1:**
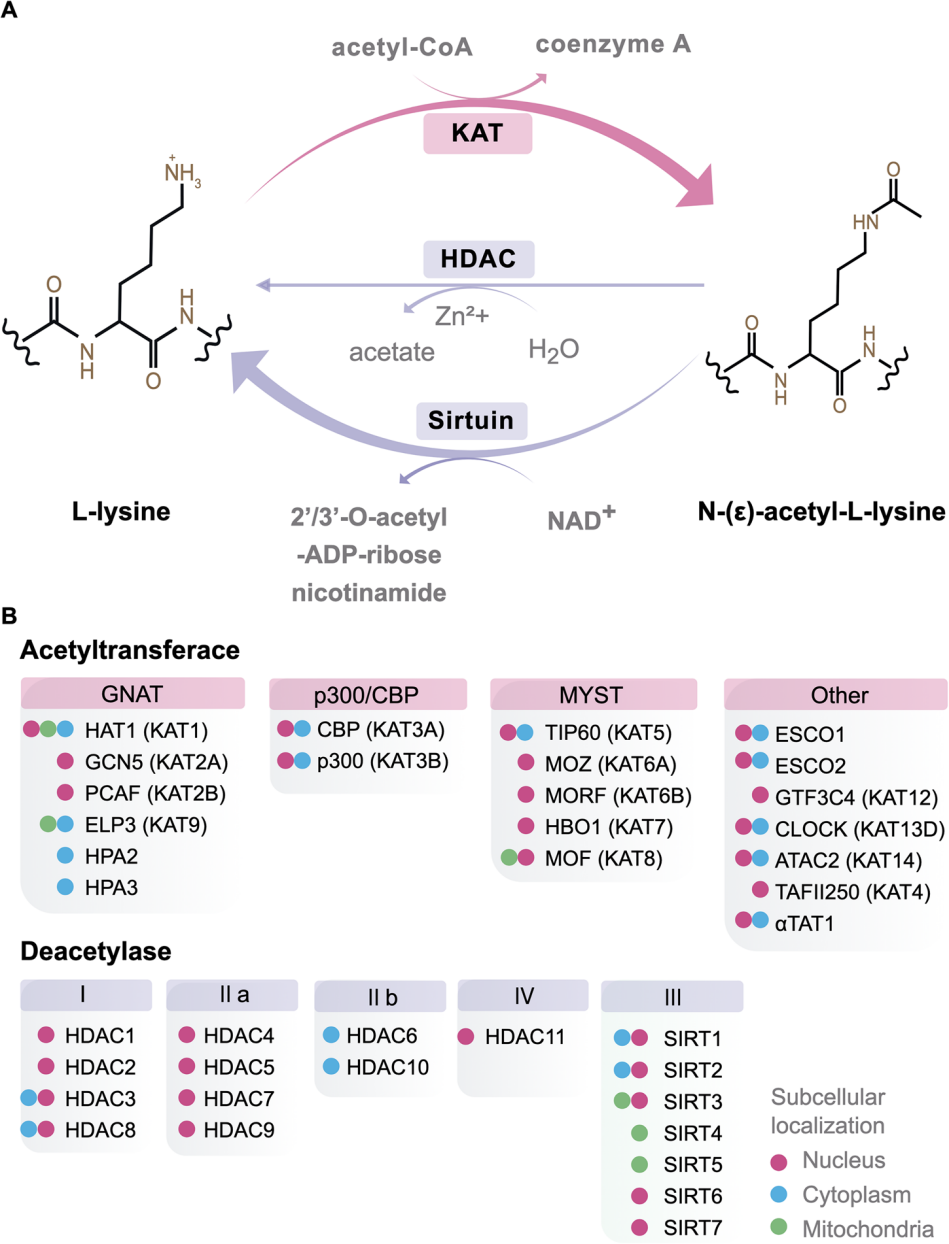
Lysine acetylation mechanism and enzyme characteristics. **A** Lysine acetylation process. **B** Classification and localization of KDAC and KAT enzymes.

Acetylation, a major form of post-translational modification (PTM), is a reversible process mediated by lysine acetyltransferases (KATs) and deacetylases (KDACs) (Fig. 1A). It involves the addition or removal of acetyl groups from the ε-amino group of lysine residues on histones and non-histone proteins [4, 10]. Dysregulated acetylation contributes to cancer development and prognosis. For instance, aberrant deacetylation promotes cancer cell proliferation, cell cycle arrest, abnormal cell death, immune disruption, immune evasion, migration, invasion, and metastasis [11, 12]. Figure 1B illustrates the classification and subcellular location of KATs and KDACs.

### Definition of autophagy and its role in cellular homeostasis

**Fig. 2:**
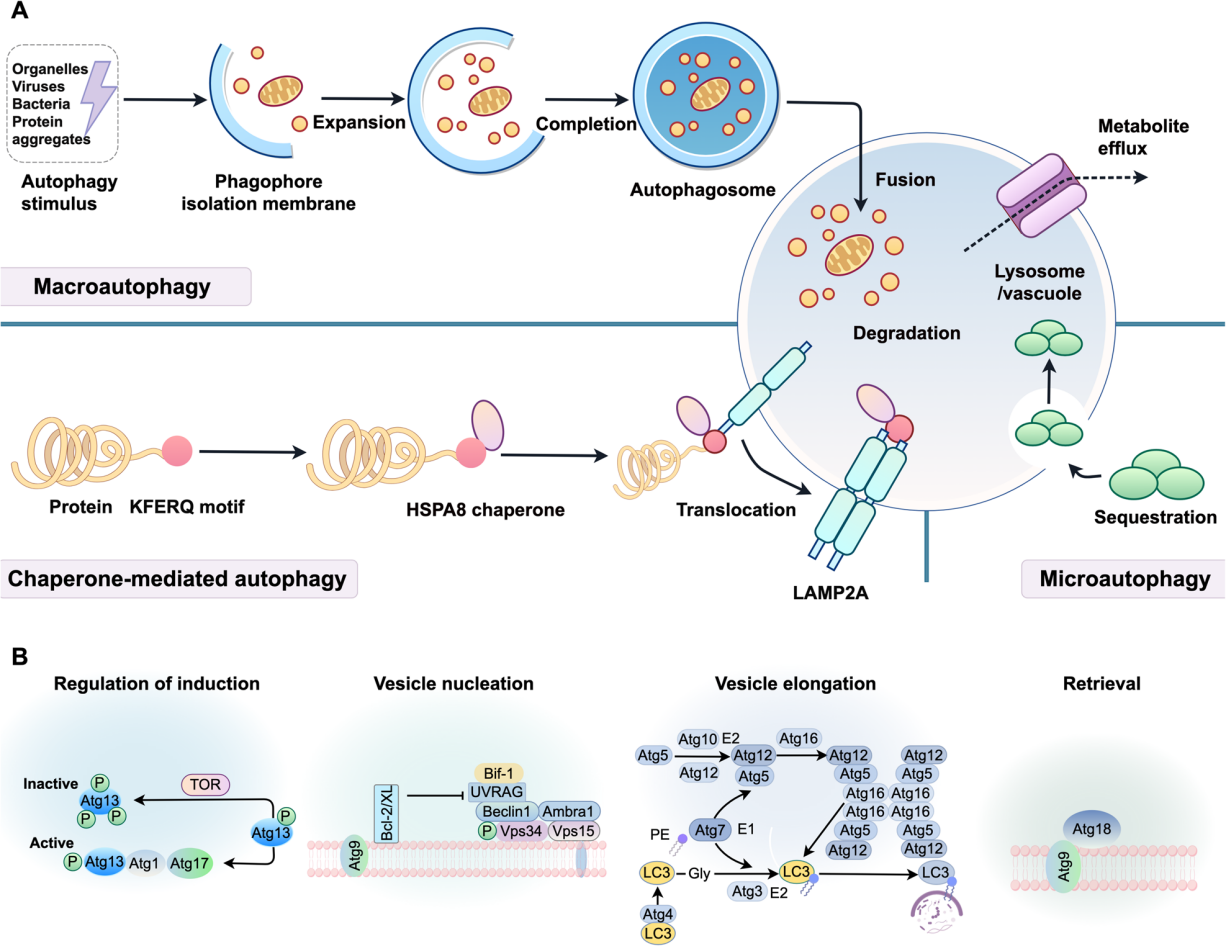
Regulatory mechanisms and molecular pathways of autophagy. **A** Subtypes of autophagy categorized by lysosomal entry pathways of cargo (substrate). **B** Key molecular components and regulatory networks underlying autophagy characterized to date.

Autophagy is a highly conserved process responsible for clearing cellular waste, including misfolded proteins and damaged organelles [13]. In mammals, autophagy occurs via three mechanisms—macroautophagy, microautophagy, and chaperone-mediated autophagy (CMA)—which differ in substrate delivery to lysosomes [14, 15]. Macroautophagy and microautophagy degrade substrates selectively or non-selectively, whereas CMA is exclusively selective (Fig. 2A). Macroautophagy involves four steps: initiation, phagophore formation, fusion with lysosomes, and degradation [16, 17], Fig. 2B details the process and regulation of macroautophagy.

## Crosstalk between acetylation and autophagy

The interaction between acetylation modifications and autophagy is a hot topic in cancer research. Acetylation regulates the function of key proteins, influencing the activation and inhibition of autophagy. Autophagy is an essential cellular stress response mechanism that helps cancer cells adapt to the external environment. The “acetylation–autophagy axis” encompasses not only the bidirectional interplay between acetylation and autophagy but also the downstream consequences of these interactions. This adaptive mechanism enables cells to respond to environmental cues, while its dysregulation is closely linked to cancer and other diseases. A clearer understanding of this axis may deepen insights into cellular networks and inform precision therapies.

### Regulation of autophagy by acetylation modifications

#### Regulation of autophagy by acetyltransferases

Acetylation modifications regulate autophagy through acetyltransferases and deacetylases, such as the HDAC family and SIRT family. Acetyltransferases play critical roles in the initiation, elongation, and maturation stages of autophagy. For instance, TIP60 initiates autophagy by acetylating ULK1 [18, 19], while p300 acetylates Beclin1 and VPS34, influencing autophagosome maturation [20, 21]. MYST1 regulates the acetylation levels of ATG7 and Beclin1, controlling the autophagic process [22], and BAG regulates p300, influencing autophagic elongation [23]. KAT5/TIP60 and Nat3 modulate autophagic cargo assembly, autophagosome transport, and fusion through the acetylation of p62 and N-terminal modifications [24].

**Fig. 3:**
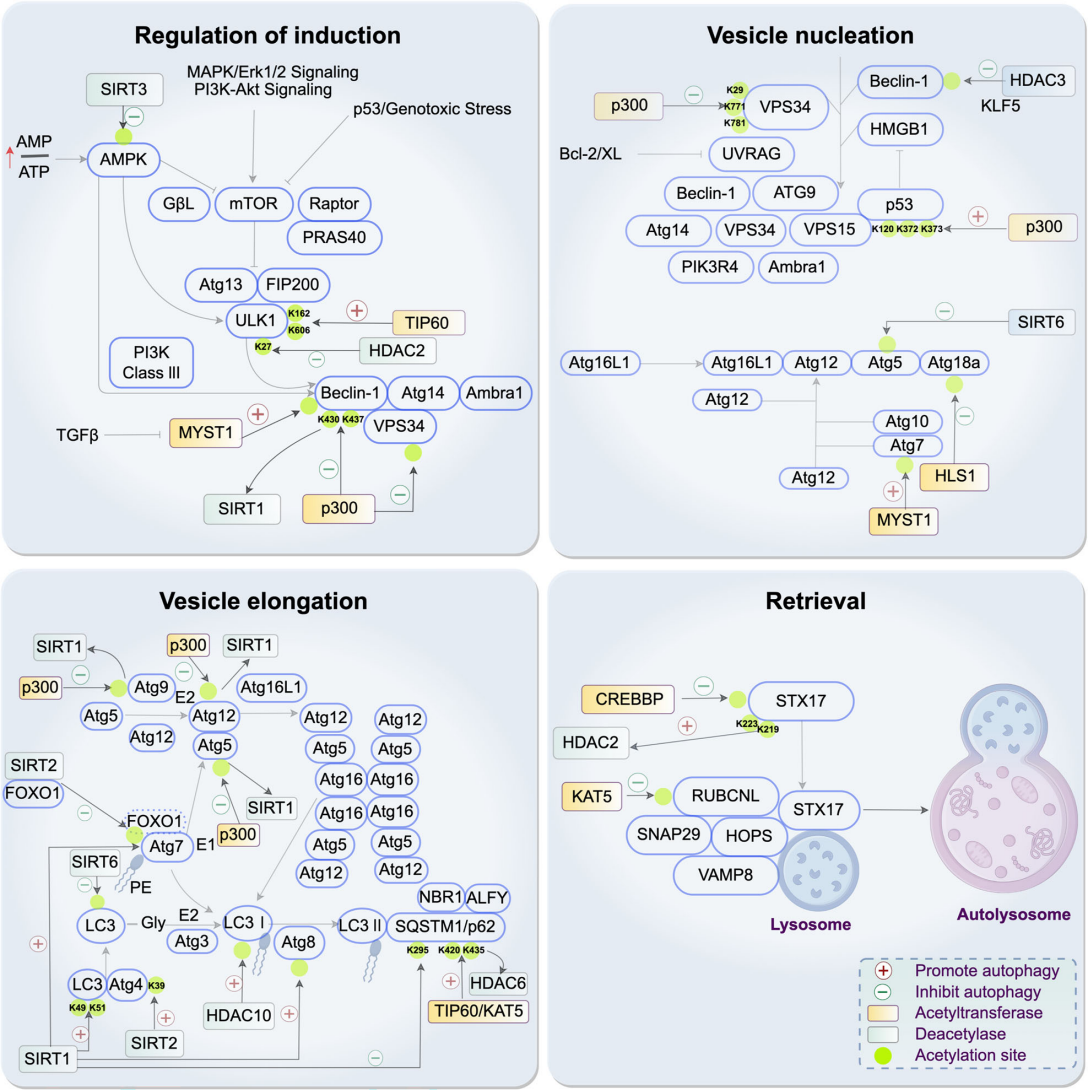
Regulatory mechanisms of acetyltransferases and deacetylases in different stages of autophagy.

Among the HDAC family, HDAC1, HDAC2, HDAC3, HDAC6, and HDAC10 regulate autophagy by affecting the expression of autophagy-related genes and protein acetylation states. For example, HDAC1 and HDAC2 negatively regulate autophagy by influencing histone acetylation and transcription factor gene expression [3, 25–27]. HDAC6 modulates autophagic recruitment [28, 29], while HDAC10 plays a role in nutrient deprivation-induced autophagy [30]. The SIRT family regulates autophagy through deacetylation of autophagy-related proteins. SIRT1 deacetylates ATG7, ATG8, and LC3 to initiate autophagy [31, 32], SIRT2 modulates autophagy through FOXO1 and ATG4B [33, 34], and SIRT3 inhibits hepatic autophagy [35]. These enzymes play complex regulatory roles at various stages of autophagy via acetylation modifications, influencing the initiation, progression, and function of autophagy (Fig. 3).

#### Regulation of autophagy by Acetyl-CoA (AcCoA)

AcCoA is a core molecule in cellular metabolism (which serves as the central metabolic node linking nutrient status to protein acetylation), acting as a key intermediary in the catabolism of fats, sugars, and proteins. It directly reflects the nutritional and energy status of the cell [36] and serves as the sole donor of acetyl groups for acetylation. Under conditions of nutrient deprivation (hunger), the levels of AcCoA rapidly decrease, leading to a reduction in the overall acetylation levels of cellular proteins. The decrease in acetylation modifications results in the deacetylation of various proteins, thereby affecting their function and activity. Depletion of AcCoA reduces the activity of the acetyltransferase P300, inducing autophagy, indicating the critical role of AcCoA in autophagy regulation [37, 38].

As previously mentioned, the autophagy process is finely regulated by energy-sensing pathways such as AMPK and nutrient-signaling pathways like mTOR. SIRT1 activates liver kinase B1 (LKB1) through deacetylation, thereby enhancing AMPK activity and promoting autophagy. When AcCoA levels decrease, AMPK is activated, and its activation inhibits mTORC1 activity, relieving its suppression of autophagy, thereby promoting the process. Conversely, when AcCoA levels rise, AMPK activity is inhibited, leading to mTORC1 activation and suppression of autophagy. However, studies have shown that in AMPK-deficient cells, changes in AcCoA levels still regulate autophagic flux (which reflects the dynamic process of autophagosome formation and degradation), suggesting that AcCoA may regulate autophagy through mechanisms independent of AMPK [39]. Further studies have revealed a negative correlation between cytosolic AcCoA levels and the phosphorylation state of mTORC1 substrate p70S6K. When AcCoA levels decrease, the phosphorylation of p70S6K increases, activating mTORC1 and inhibiting autophagy; whereas when AcCoA levels rise, p70S6K phosphorylation decreases, inhibiting mTORC1 activity and promoting autophagy initiation. This finding further confirms that AcCoA regulates autophagy not only through the AMPK/mTORC1 signaling pathway but also possibly through AMPK-independent mechanisms [39, 40].

### Regulation of acetylation by autophagy

#### Degradation of acetyltransferases and deacetylases mediated by autophagy

The degradation of acetyltransferases and deacetylases is crucial for cellular function and metabolic regulation. Autophagy influences the activity and regulatory roles of these enzymes through their selective degradation. Autophagy specifically degrades the acetyltransferase P300, thereby affecting the acetylation status of FOXO1. The normal function of P300 is to acetylate FOXO1, preventing its nuclear entry. When autophagy is activated, P300 is degraded, resulting in FOXO1 deacetylation, enabling its translocation to the nucleus where it binds to the promoter region of MIR449A (1461–1471 bp), promoting its transcription. By regulating the expression of MIR449A, this process affects cellular functions, potentially driving the progression of related diseases [41].

Deacetylase can also be degraded by autophagy. Studies have shown that increased histone acetylation usually leads to chromatin relaxation, thereby activating the transcription of related genes. Pharmacological or genetic inhibition of autophagy prevents the degradation of HDACs, indicating that autophagy specifically targets and degrades HDACs to regulate gene transcription and cellular functions [7]. Furthermore, it has been found that treatment with drugs that enhance autophagosome formation results in an upregulation of autophagy and a significant reduction in the protein levels of HDACs, particularly cytosolic HDAC6, further supporting the essential role of autophagy in the degradation of acetyltransferases and deacetylases [42, 43]. Autophagy also regulates the expression of deacetylase SIRT1 during cellular senescence by selectively degrading SIRT1. In the aging process, dephosphorylation of SIRT1 may serve as a signal for its degradation. SIRT1 is a nuclear substrate of autophagy, and its interaction with LC3 is considered a key mechanism for autophagy-mediated degradation of SIRT1. In senescent cell models, the downregulation of SIRT1 is closely associated with autophagy activation, affecting cellular metabolism and stress responses, thus regulating cellular functions during aging [44, 45]. Additionally, the degradation of SIRT3 also occurs through selective autophagy, especially under stress conditions, such as mitochondrial dysfunction. Studies have shown that during Newcastle disease virus (NDV) infection, mitochondrial dysfunction leads to excessive mitochondrial reactive oxygen species (mROS), triggering cellular stress. To cope with this stress, cells use the PINK1-PRKN-dependent autophagic pathway to clear damaged mitochondria, during which SIRT3 is degraded. This process not only reduces the activity of SIRT3 but also enhances the stability of HIF-1A and the activity of glycolytic metabolism, influencing cellular stress responses [46].

#### Regulation of Acetyl-CoA levels by autophagy

Studies have shown that autophagy plays a crucial role in AcCoA synthesis. Autophagy indirectly promotes the synthesis of AcCoA by providing citrate, a precursor of AcCoA metabolism. Cancer cells rely on autophagy rather than directly activating mitochondrial function or enhancing mitochondrial quality to provide the citrate required for AcCoA synthesis. Although citrate synthesis can occur via both mitochondrial-dependent and independent pathways, in the absence of autophagy, mitochondria still maintain some function, indicating that autophagy plays a dominant role in AcCoA synthesis. Inhibitors of autophagy-related enzymes CAMKK2 and ACLY can interfere with the synthesis of AcCoA through autophagy. Activation of autophagy promotes the increase of AcCoA, which further enhances the acetylation of TFEB, forming a positive feedback loop that activates autophagy [47, 48]. TFEB is a key regulatory factor in autophagy and lysosomal function, and its transcriptional activity is typically negatively regulated by mTORC1. Phosphorylation by mTORC1 causes TFEB to remain in the cytoplasm and inhibits its transcriptional activity. Acetylation, as a post-translational modification, can enhance TFEB stability, increase its nuclear levels and activity, and further promote autophagy [48].

### Bidirectional feedback between acetylation and autophagy

**Fig. 4:**
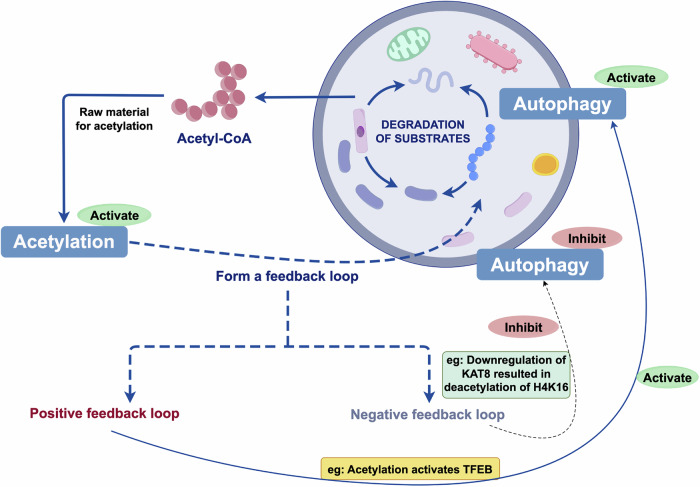
Bidirectional feedback between acetylation and autophagy.

In addition to unidirectional regulation, acetylation and autophagy also engage in multilayered bidirectional feedback. At the metabolic level, autophagy provides substrates for acetylation by generating acetyl-CoA, while acetylation in turn regulates autophagy through the transcription of autophagy-related genes and the functional modulation of autophagy proteins. Under nutrient-rich conditions, acetyl-CoA accumulation promotes acetylation, activates mTOR, and suppresses autophagy; conversely, under energy-deprived conditions, autophagy induction lowers acetyl-CoA levels and activates SIRT1-dependent deacetylation of core proteins, thereby enhancing autophagy [3, 47, 49, 50]. Deacetylases such as SIRT1, SIRT3, and SIRT6 further exemplify this interdependence: they promote autophagy via deacetylation while relying on the metabolic homeostasis maintained by autophagy to sustain their own activity [51]. At the epigenetic level, autophagy-induced downregulation of KAT8 leads to H4K16 deacetylation, thereby repressing transcription of autophagy-related genes, which not only prevents excessive activation of autophagy but also influences cell fate. In later stages of autophagy, reduced MOF expression and activation of SIRT1 decrease acetylation levels and terminate autophagy, forming a negative feedback loop [52–54]. Similar regulatory circuits exist in specific signaling pathways. For example, IKKα promotes p53 acetylation to induce autophagy, while autophagy in turn degrades IKKα [55]. Acetylation of GRP78 upregulates VPS34 and induces autophagy, whereas VPS34 enhances GRP78 stability [56]. In lung cancer cells, autophagy promotes acetyl-CoA accumulation, which enhances the acetylation of Snail and TFEB, thereby amplifying autophagy and metastatic potential [48]. Collectively, acetylation and autophagy form both positive and negative feedback loops through metabolic supply, deacetylase regulation, epigenetic modification, and signaling crosstalk, playing essential roles in cellular homeostasis and disease progression (Fig. 4).

## Roles of acetylation-autophagy crosstalk in cancer

Mounting evidence indicates that the interplay between acetylation and autophagy extends beyond basic cellular homeostasis and plays crucial roles in cancer biology. This crosstalk influences multiple hallmarks of cancer, including tumor initiation, progression, drug resistance, prognosis, and interactions with the tumor microenvironment (Table 1).

**Table 1:** Acetylation-mediated regulation of autophagy across cancer types and subtypes.

Cancer Type	Subtype / Context	Acetyltransferase/Deacetylase	Target	Acetylation Site	Effect on Autophagy	Tumor Impact	Ref.
Breast Cancer	ER⁺ and TNBC	-/HDAC6	MST1	K35	MST1 deacetylation promotes its degradation via CMA	Promote proliferation and survival	[9]
Breast Cancer	ER⁺ Luminal A, ER⁺/HER2⁺ Luminal B, HER2⁺	CREBBP/-	RB1CC1	K276	RB1CC1 acetylation promotes macroautophagy	-	[75]
Breast Cancer	ER⁺, Luminal A	CREBBP/SIRT3	HSD17B4	K669	HSD17B4 acetylation promotes its degradation via CMA	Inhibit migration and invasion	[68]
Breast Cancer	Luminal A Luminal B TNBC	p300/-	HOXB13	K277	HOXB13 acetylation inhibits its degradation via CMA	Promote cell growth and drug resistance	[60]
Colorectal Cancer	MSI, MSS	-/SIRT1	Histone H4	K16	Histone H4 acetylation inhibits macroautophagy	Enhance migration and invasion	[73]
Colorectal Cancer	MSS MSI-H	-/SIRT3	SHMT2	K95	SHMT2 acetylation promotes its degradation via macroautophagy	Inhibits cell proliferation and tumor growth	[65]
Colorectal Cancer	MSI-H	-/SIRT5	LDHB	K329	LDHB deacetylation promotes macroautophagy	Promote tumor growth	[64]
Colorectal Cancer	MSS MSI-H	-/SIRT7	RAN	-	RAN acetylation inhibits macroautophagy	Promote proliferation, invasion, and metastasis	[74]
Prostate Cancer	AR+	-/HDACs	Histone H3	K9, K27	Histone H3 acetylation promotes macroautophagy	-	[85]
Prostate Cancer	AR+AR-	CREBBP/SIRT3	HSD17B4	K669	HSD17B4 acetylation promotes its degradation via CMA	Inhibit proliferation and invasion	[69]
Prostate Cancer	AR+AR-	KAT2B/HDAC2	TPD52	K163	TPD52 deacetylation promotes CMA	Promote tumor growth	[78]
Prostate Cancer	AR+AR-	PCAF/HDACs	δ-catenin	-	δ-catenin acetylation promotes its degradation via macroautophagy	Inhibit proliferation and migration	[72]
Hepatocellular Carcinoma	-	-/HDAC6	α-tubulin	-	α-tubulin acetylation promotes macroautophagy	Suppress tumorigenesis	[59]
Hepatocellular Carcinoma	-	-/SIRT1	FOXO3	-	FOXO3 deacetylation promotes mitophagy	Promote mitochondrial renewal and cancer cell survival	[86]
Hepatocellular Carcinoma	-	GCN5/SIRT1	p62	K295	Inhibit autophagy activity	Promote proliferation, survival, and invasion	[57]
Melanoma	-	-/SIRT1	Beclin 1	K430, K437	Beclin 1 deacetylation promotes macroautophagy	Promote EMT, invasion, and metastasis	[128]
Melanoma	-	p300/HDAC10	Histone H3	K27	Histone H3 acetylation promotes macroautophagy	Inhibit proliferation and enhance drug sensitivity	[79]
Cervical Cancer	-	ACAT1/HDAC2	Parkin	K129, K220, K349	Parkin acetylation promotes mitophagy	Inhibit proliferation and growth	[71]
Gastric Cancer	-	-/SIRT1	ATG2B	-	ATG2B acetylation promotes macroautophagy	Promote proliferation and invasion	[70]
Glioblastoma	-	ARD1/-	PGK1	K388	PGK1 acetylation promotes macroautophagy	Promote proliferation and tumorigenesis	[81]
Lung Cancer	NSCLC	-/-	TFEB	-	TFEB acetylation promotes macroautophagy	Promote EMT, invasion, and metastasis	[48]
Pancreatic Cancer	PDAC	-/SIRT2	LDHA	K5	LDHA acetylation promotes its degradation via CMA	Inhibit proliferation and migration	[80]

### Roles of acetylation–autophagy crosstalk in tumor initiation

The crosstalk between autophagy and acetylation modifications plays a crucial role in the initiation of cancer. In hepatocellular carcinoma (HCC), SIRT1 deacetylates p62 at K295, preventing its degradation, activating mTORC1, and promoting hepatocarcinogenesis [57], while PCAF enhances autophagic flux by inhibiting Akt/mTOR, thereby inducing cancer cell death [58]. Loss of RASSF1A reduces microtubule acetylation and impairs autophagosome trafficking, promoting oxidative stress and liver cancer development, whereas restoring RASSF1A or enhancing autophagy suppresses tumorigenesis [59]. In breast cancer, HBXIP recruits p300 to acetylate HOXB13 at K277, stabilizing the protein and preventing its CMA-mediated degradation, which drives ERα-dependent proliferation [60, 61], while acetylation of MST1 stabilizes its tumor-suppressive role in the Hippo pathway, and its dysregulation facilitates cancer initiation [62]. In colorectal cancer (CRC), PINK1-mediated mitophagy reduces AcCoA levels to suppress metabolic reprogramming and tumor growth, while elevated AcCoA reverses this effect [63]. SIRT5 deacetylates LDHB at K329, enhancing its activity and autophagy, thereby promoting CRC progression [64]. Acetylation of SHMT2 at K95 disrupts its structure and function, leading to lysosomal degradation, reduced serine metabolism, and suppressed proliferation; autophagy contributes by degrading acetylated SHMT2 to limit cancer growth [65]. Furthermore, acetylation of TFEB enhances its stability and nuclear activity, sustaining autophagic flux despite mTORC1 repression, which promotes tumor growth and metastasis [48, 66]. Collectively, these findings highlight how acetylation–autophagy interplay fine-tunes oncogenic signaling, metabolism, and transcriptional programs, driving tumor initiation and early development.

### Roles of acetylation–autophagy crosstalk in tumor progression

The crosstalk between acetylation modifications and autophagy plays an essential role in cancer progression. In melanoma, fatty acid oxidation (FAO) regulates autophagosome formation via AcCoA, promoting cell migration and invasion, whereas FAO inhibition increases autophagosomes but suppresses invasion [67]. SIRT1 deacetylates Beclin1 to promote E-cadherin degradation and EMT, thereby enhancing melanoma invasiveness, which can be reversed by autophagy inhibition [67]. In breast and prostate cancers, CREBBP and SIRT3 regulate HSD17B4 acetylation at K669, which facilitates HSPA8-mediated CMA degradation, reducing HSD17B4 accumulation and suppressing migration and invasion [68, 69]. In gastric cancer, SIRT1 deacetylates ATG2B, enhancing its RNF5-mediated ubiquitination and degradation, thereby suppressing autophagy and promoting tumor progression [70]. In cervical cancer, ACAT1 acetylates Parkin to promote mitophagy and inhibit proliferation, whereas HDAC2 deacetylates Parkin to block its function and accelerate progression [71]. In prostate cancer, PCAF acetylates δ-catenin to induce autophagic degradation and inhibit growth, while HDAC stabilizes δ-catenin via deacetylation, promoting proliferation [72]. In CRC, Ube2v1 suppresses SIRT1 activity, reducing H4K16 acetylation and autophagy gene expression, thereby enhancing EMT-driven invasion [73]. Similarly, RSL1D1 inhibits SIRT7, increases RAN acetylation, promotes STAT3 nuclear accumulation, represses autophagy-related genes, and drives CRC progression [74]. Collectively, these findings highlight the multifaceted role of acetylation–autophagy crosstalk in facilitating tumor progression.

### Roles of acetylation–autophagy crosstalk in tumor drug resistance

The crosstalk between acetylation modification and autophagy plays a critical role in cancer drug resistance. The crosstalk between acetylation and autophagy plays a critical role in cancer drug resistance. In breast cancer, CREBBP acetylates RB1CC1 at K276, preventing its ubiquitination and degradation, thereby stabilizing RB1CC1, enhancing autophagy, and promoting resistance under stress conditions [75]. Meanwhile, HBXIP activates NF-κB to upregulate HDAC6, which deacetylates MST1 at K35, disrupts Hippo signaling, and accelerates MST1 degradation via CMA, ultimately driving resistance to tamoxifen [9]. In triple-negative breast cancer, H3K27 acetylation upregulates DDIT4-AS1, which activates autophagy and confers paclitaxel resistance, while aberrant HDAC activity further highlights the role of acetylation–autophagy crosstalk in therapy resistance [76, 77]. In castration-resistant prostate cancer, acetylated TPD52 interacts with HSPA8 to activate CMA and suppress macroautophagy, enabling tumor cells to withstand therapy-induced stress [78]. In melanoma, HDAC10 cooperates with p300 to regulate H3K27 acetylation and SPARC transcription; its depletion induces autophagy, activates AMPK signaling, and reduces drug resistance [79]. Collectively, these findings demonstrate that acetylation–autophagy interplay critically influences tumor resistance, which has potential as a therapeutic target to provide a basis for therapeutic strategies.

### Roles of acetylation–autophagy crosstalk in tumor prognosis

The interaction between acetylation modification and autophagy is of significant importance in tumor prognosis. The crosstalk between acetylation and autophagy significantly influences tumor prognosis. In liver cancer, PCAF-mediated H4 acetylation enhances autophagy and is associated with better outcomes [58]. In cervical cancer, ACAT1 acetylates Parkin to promote mitophagy and maintain mitochondrial homeostasis, correlating with favorable prognosis [71]. In pancreatic cancer, LDH-A K5 acetylation triggers CMA-mediated degradation, while reduced acetylation stabilizes LDH-A, enhances glycolysis, and predicts poor prognosis [80]. In glioblastoma, ARD1 acetylates PGK1 at K388 to initiate autophagy, correlating with higher malignancy and poor prognosis [81]. In colorectal cancer, Ube2v1 inhibits Sirt1, reduces H4K16 acetylation, suppresses autophagy, and promotes EMT and invasion, indicating unfavorable prognosis [73]. In gastric cancer, SIRT1-mediated deacetylation of ATG2B enhances autophagy and invasiveness, correlating with poor prognosis [70]. These findings highlight the prognostic value of acetylation–autophagy crosstalk.

### Roles of acetylation–autophagy crosstalk in the tumor microenvironment

The crosstalk between acetylation modification and autophagy plays a crucial role in the tumor microenvironment, influencing tumor invasion, metastasis, and immune evasion by regulating intracellular and extracellular metabolism and signaling pathways. Within the TME, tumor cells adapt to cues such as hypoxia, nutrient deprivation, and stromal signals via dynamic acetylation–autophagy interactions. In lung cancer, extracellular SIRT2 from autophagosomes deacetylates ECM proteins (e.g., integrin β3), disrupting cell–matrix interactions and promoting metastasis [82]. In gastric cancer peritoneal metastasis, hypoxia-induced autophagy degrades SIRT1, increasing HIF-1α acetylation and VEGFA signaling, enhancing adhesion and invasion [83]. In glioblastoma, TME metabolic stress triggers ARD1-mediated PGK1 acetylation, promoting autophagosome formation and tumor survival [81, 84]. In prostate cancer, hypoxia suppresses autophagy, elevating 3βHSD1 and androgen synthesis, while HDAC inhibition restores autophagy and limits progression [85]. In liver cancer, CDK9 inhibition blocks mitophagy via the SIRT1–FOXO3–BNIP3 axis, inducing mitochondrial dysfunction and cell death under TME metabolic stress [86].

The crosstalk between acetylation and autophagy also profoundly shapes immune cell functions by modulating metabolic substrate supply, epigenetic modifications, and signaling pathways, thereby determining efficacy and persistence. Autophagy coupled with histone deacetylation modulates CD8⁺ T cell effector function and preserves stemness [87], while EP300 inhibition by aspirin induces autophagy to enhance CD8⁺ T cell immunity [88]. Tumor cell autophagy remodels ATP/adenosine metabolism, strengthening CD8⁺ T cell responses and reducing Treg recruitment [89]. In antigen-presenting cells, autophagy regulates cGAS acetylation, promoting type I IFN and PD-L1 expression [90], and autophagy-mediated HDAC inhibition activates macrophages via HMGB1 acetylation [91]. In Tregs, caloric restriction lowers acetylation and activates autophagy, reducing immunosuppression [92, 93]. In tumor-infiltrating T cells, reduced acetyl-CoA triggers autophagy and epigenetic reprogramming, enhancing stem-like cytotoxicity and immunotherapy efficacy [94]. Taken together, acetylation–autophagy interactions reshape immune cell function through multi-layered mechanisms, directly influencing tumor immune surveillance and immune evasion (Table 2).

**Table 2:** Immune cell–intrinsic effects of acetylation–autophagy crosstalk in the tumor microenvironment.

Immune Cell Type	Cancer Context	Representative Acetylation–Autophagy Interaction	Effect on Immune Cell Function	Tumor-Related Outcome	Ref.
CD8⁺ T cells	Solid tumor TME with high extracellular K⁺	High K⁺ induces autophagy and reduces histone acetylation	Effector function suppressed; stemness and persistence maintained	Improved tumor clearance	[87]
CD8⁺ T cells (T lymphocytes)	Solid tumors with immunogenic chemotherapy	Aspirin inhibits EP300, decreases acetylation, induces autophagy	Enhanced T cell-mediated immunity	Better tumor control	[88]
CD8⁺ T cells / Treg	Transplantable and induced tumors	Tumor-cell autophagy alters ATP/adenosine metabolism	CD8⁺ response increased; Treg recruitment reduced	Reduced tumor growth	[89]
Dendritic cells / Myeloid cells	Multiple cancers	Autophagy regulates cGAS acetylation	Increased type I IFN and PD-L1 expression	Both antitumor immunity and immune evasion	[90]
Macrophages	Cancer and ferroptosis models	Autophagy-mediated HDAC inhibition leads to HMGB1 acetylation and release	HMGB1 activates macrophages	Immune activation in TME	[91]
Regulatory T cells (Tregs)	KRAS-driven lung cancer (mouse model)	CRMs decrease lysine acetylation, activate autophagy	Treg depletion, reduced immunosuppression	Enhanced immunosurveillance, reduced tumor mass	[93]
Regulatory T cells (Tregs)	Tumors under caloric restriction or treated with CRMs	Caloric restriction reduces acetylation, activates autophagy	Reduced Treg infiltration	Improved immune control and therapy response	[92]
Tumor-infiltrating T cells (CD8⁺)	Solid tumors with caloric-restricted state	Decreased acetyl-CoA induces autophagy and epigenetic reprogramming	Acquisition of stem-like phenotype, cytotoxicity preserved	Improved antitumor activity and immunotherapy response	[94]

### Comparative perspectives across cancer types and subtypes

Distinct cancers rely on unique regulatory circuits: for instance, HCC frequently engages SIRT1–p62 or PCAF–microtubule pathways, whereas breast cancer emphasizes p300/CREBBP-driven stabilization of transcription factors (e.g., HOXB13, RB1CC1). In colorectal cancer, metabolic enzymes such as SHMT2, LDHB, and TFEB are selectively targeted through acetylation-dependent degradation or stabilization, reflecting the metabolic vulnerability of this tumor type. Importantly, tumor heterogeneity further shapes this crosstalk. In breast cancer, ER⁺ luminal versus TNBC subtypes exhibit distinct reliance on acetylation-regulated autophagy: luminal tumors often depend on ER–p300 signaling, whereas TNBC exploits H3K27 acetylation and DDIT4-AS1–driven autophagy to resist chemotherapy. Similarly, in colorectal cancer, MSI-H tumors are characterized by SIRT5/LDHB-dependent metabolic control, while MSS tumors show stronger regulation by chromatin modifiers such as SIRT1 and SIRT7. Prostate cancer also demonstrates subtype dependence, with androgen receptor (AR)-positive versus AR-independent contexts engaging different acetylation–autophagy axes (e.g., PCAF/δ-catenin versus TPD52–CMA). Together, the acetylation–autophagy axis is not uniform across cancers but instead shaped by tissue origin and molecular subtype (Table 1).

## Potential of targeting acetylation and autophagy for cancer therapy

### Targeting acetylation in cancer therapy

The research and application of acetylation-related drugs have become a hotspot in cancer therapy. As a key PTM, acetylation regulates the acetylation status of both histone and non-histone proteins, and is extensively involved in various cellular processes such as gene expression, cell cycle progression, and DNA damage repair [95]. Disruption of the balance between acetylation and deacetylation can lead to dysregulated gene expression, thereby promoting tumor initiation and progression [96]. Therefore, the development of drugs targeting acetylation-related enzymes—such as KATs and KDACs—has provided new strategies for cancer treatment.

HDAC inhibitors increase the acetylation levels of histone and non-histone proteins by inhibiting HDAC activity, thereby altering gene expression and cellular processes. Multiple HDAC inhibitors have demonstrated promising anti-tumor efficacy in clinical trials [97, 98]. For instance, drugs such as Vorinostat, Romidepsin, and Belinostat have been approved by the FDA for treating certain types of lymphomas. Moreover, combining HDAC inhibitors with other therapies, including chemotherapy and immunotherapy, is also being actively investigated [99, 100]. For example, HDAC6 inhibitors used in combination with PD-L1 antibodies can enhance the anti-tumor function of γδ T cells [101]. Various HDAC inhibitors listed in Table 3, such as Entinostat (MS-275) and Panobinostat (LBH589), are currently undergoing clinical trials for a range of cancers, including breast cancer, non-small cell lung cancer, and leukemia, demonstrating their broad therapeutic potential.

**Table 3:** Clinical trials of cancer therapy targeting acetylation.

Class	Compound	Alias	Target	Most advanced clinical trials	Phase	Combination therapy	Cancer Type
HDAC inhibitor	NSC 696085	Pyroxamide	HDAC1	NCT00042900	I	None	Advanced Malignancies
	TNG260	/	HDAC1	NCT05887492	I/II	Pembrolizumab	Advanced Solid Tumors
	VPA	Valproic acid	HDAC1	NCT03048084	IV	None	Glioma
	FK228	Romidepsin	HDAC1/2	NCT01796002	III	CHOP	Peripheral T Cell Lymphoma
	4SC-202	Domatinostat	HDAC1/2/3	NCT04393753	II	Avelumab	Merkel Cell Carcinoma
	CI-994	Tacedinaline	HDAC1/2/3	NCT00005093	III	Gemcitabine	Lung Cancer
	CXD101	Zabadinostat	HDAC1/2/3	NCT05873244	II	Geptanolimab	Liver Cancer
	MS-275	Entinostat	HDAC1/2/3	NCT02115282	III	Exemestane	Breast Cancer
	Chidamide	Tucidinostat	HDAC1/2/3/10	NCT05191914	IV	Fulvestrant	Breast Cancer
	CUDC-907	Fimepinostat	HDAC1/2/3/10	NCT01742988	I	Rituximab, Venetoclax	Lymphoma
	MGCD0103	Mocetinostat	HDAC1/2/3/11	NCT00372437	I/II	Gemcitabine	Refractory solid tumors
	ACY-241	Citarinostat	HDAC1/2/3/6/8	NCT02551185	I	Paclitaxel	Advanced Solid Tumors
	JNJ-26481585	Quisinostat	HDAC1/2/4/10/11	NCT02948075	II	Paclitaxel, Carboplatin	Ovarian Cancer
	ITF2357	Givinostat	HDAC1/3	NCT00792506	II	None	Multiple Myeloma
	RAS2410	Resminostat	HDAC1/3/6	NCT00943449	II	Sorafenib	Hepatocellular Carcinoma
	ABR-215050	Tasquinimod	HDAC4	NCT01234311	III	None	Prostate Cancer
	ACY-1215	Ricolinostat	HDAC6	NCT01323751	I/II	Bortezomib, Dexamethasone	Multiple Myeloma
	KA2507	/	HDAC6	NCT03008018	I	None	Solid Tumors
	Phenylacetate	Phenylacetic acid	HDAC6	NCT00003241	II	None	Brain and Central Nervous System Tumors
	4-PBA	Phenylbutyrate	HDACs	NCT00006450	II	None	Brain Tumor
	AN-9	Pivanex	HDACs	NCT00073385	II	Docetaxel	Non-Small-Cell Lung
	AR-42	HDAC-42	HDACs	NCT01798901	I	Decitabine	Acute Myeloid Leukemia
	CHR-2845	Tefinostat	HDACs	NCT02759601	I/II	None	Hepatocellular Carcinoma
	CUDC-101	/	HDACs	NCT00728793	I	None	Advanced Solid Tumors
	EDO-S101	Tinostamustine	HDACs	NCT03345485	I/II	None	Advanced Solid Tumors.
	LBH589	Panobinostat	HDACs	NCT02386800	IV	Ruxolitinib	Leukemia
	PCI-24781	Abexinostat	HDACs	NCT03592472	III	Pazopanib	Renal Cell Carcinoma
	PXD101	Belinostat	HDACs	NCT06072131	III	CHOP	Peripheral T Cell Lymphoma
	SAHA	Vorinostat	HDACs	NCT00773747	III	Bortezomib	Multiple Myeloma
	SB939	Pracinostat	HDACs	NCT03151408	III	Azacitidine	Acute Myeloid Leukemia
	SHP-141	Remetinostat	HDACs	NCT03180528	II	None	Skin Basal Cell Carcinoma
	Sulforaphane	/	HDACs	NCT03232138	II	None	Lung Cancer
	TSA	Trichostatin A	HDACs	NCT03838926	I	None	Relapsed or Refractory Hematologic Malignancies
Sirtuin Activator	Fisetin	/	SIRT1	NCT05595499	II	None	Breast Cancer
	Quercetin	Sophoretin	SIRT1	NCT06355037	II	Dasatinib	Breast Cancer
	SRT501	Resveratrol	SIRT1	NCT03253913	II	Sirolimus	Lymphangioleiomyomatosis
Sirtuin Inhibitor	Niacinamide	Vitamin PP	SIRT1/2	NCT00033436	III	Carbogen, Radiation therapy	Bladder Cancer
	Suramin	/	SIRT1/2/5	NCT00002723	III	None	Prostate Cancer
HAT Inhibitor	PRI-724	Foscenvivint	CBP	NCT01606579	I/II	None	Advanced Myeloid Malignancies
	Procyanidin	Proanthocyanidins	p300	NCT03465345	I	Metformin	Prostate Cancer
	CCS1477	Inobrodib	p300/CBP	NCT04068597	I/II	Pomalidomide, Dexamethasone, Azacitidine, Venetoclax	Advanced Hematological Malignancies
	Curcumin	Diferuloylmethane	p300/CBP	NCT02064673	III	None	Prostate Cancer
	NEO2734	EP31670	p300/CBP	NCT05488548	I	None	Advanced Solid Tumors and Hematological Malignancies
	Pentamidine	/	Tip60	NCT00809796	I/II	None	Colorectal Cancer

Sirtuins also play critical roles in gene expression, DNA repair, and metabolic regulation. Their functions in cancer are dualistic: certain SIRTs (e.g., SIRT1) can promote tumor progression in some cancers, while others (e.g., SIRT6) act as tumor suppressors by maintaining genomic stability. In recent years, significant progress has been made in the development of small-molecule activators and inhibitors targeting Sirtuins. For instance, SIRT3 regulates mitochondrial function via deacetylation and has been implicated in endocrine therapy resistance in breast cancer [102]. Additionally, SIRT1 activators such as resveratrol have shown anti-tumor potential in preclinical cancer models [103]. Table 3 also lists related clinical trials of Sirtuin activators and inhibitors, such as Resveratrol (SRT501) and Niacinamide, which are being investigated for the treatment of lymphangioleiomyomatosis and bladder cancer, respectively.

HATs such as CBP/p300 also play important roles in cancer. CBP/p300 catalyzes the acetylation of histone H3K27, thereby activating transcription of various oncogenes and promoting tumor cell proliferation and survival [104]. In recent years, small-molecule inhibitors and degraders targeting CBP/p300 (e.g., CCS1477 and XYD190) have been developed. These compounds inhibit tumor growth and overcome chemotherapy resistance by downregulating oncogene expression. As mentioned in Table 3, CCS1477 (Inobrodib) is undergoing clinical trials for the treatment of advanced hematologic malignancies, demonstrating its clinical potential as a HAT inhibitor.

Combination therapy involving acetylation-related drugs offers new directions for cancer treatment. For example, combining HDAC inhibitors with immune checkpoint inhibitors can enhance the efficacy of immunotherapy. Furthermore, the co-administration of CBP/p300 inhibitors with chemotherapeutic agents can significantly improve anti-tumor effects. These combination strategies not only enhance treatment outcomes but also provide new approaches to overcome therapeutic resistance.

### Targeting autophagy in cancer therapy

Targeting autophagy has become a research hotspot in cancer therapy in recent years. Autophagy plays a crucial role in cellular physiology by degrading and recycling damaged organelles and proteins to maintain cellular homeostasis. However, in cancer, autophagy exhibits significant duality: on the one hand, autophagy can serve as a protective mechanism, helping cancer cells survive under metabolic stress, hypoxia, or therapy-induced stress conditions; on the other hand, excessively activated autophagy may lead to cell death [3, 105, 106]. This complex biological characteristic makes targeting autophagy a promising cancer therapy strategy, while also presenting many challenges.

The activation of autophagy in cancer cells is usually closely related to their rapid proliferation and metabolic demands. Cancer cells activate autophagy to maintain energy supply, enhance stress tolerance, limit damage, and prevent cell death [107, 108]. Most cancer treatments, including radiotherapy, chemotherapy, and targeted therapy, can induce autophagic responses [109]. However, this autophagic response often becomes a defense mechanism for cancer cells, allowing them to enter a dormant state, thereby leading to treatment resistance and disease recurrence [110]. Therefore, inhibiting autophagy is considered a potential strategy to enhance the efficacy of anti-cancer therapies. Chloroquine (CQ) and hydroxychloroquine (HCQ) are currently the only FDA-approved autophagy inhibitors for cancer treatment. They interfere with lysosomal acidification, inhibit the fusion of autophagosomes with lysosomes, and thus block the autophagy process [111, 112]. Multiple studies have shown that CQ/HCQ can enhance the sensitivity of cancer cells to chemotherapeutic drugs and even reverse chemotherapy resistance. For example, in clinical trials of pancreatic cancer and glioma, the combination of CQ/HCQ with RAS pathway inhibitors or BRAF inhibitors demonstrated more effective anti-tumor effects than monotherapy [96]. However, the clinical application of CQ/HCQ still faces challenges, partly due to their limited specificity towards the autophagy pathway, resulting in limited therapeutic efficacy. Moreover, the role of autophagy in cancer is highly context-dependent. In some cases, autophagy may promote tumor growth, while in others it may suppress it. For instance, certain drugs (such as gemcitabine) exert anti-tumor effects through autophagy activation, indicating that in some cases, activating rather than inhibiting autophagy may be a more effective therapeutic strategy.

Table 4 summarizes the current clinical trials targeting autophagy, covering various targeted drugs and their applications in different types of cancer. These trials involve a variety of autophagy-targeting agents, including AMPK, mTOR, and PI3K/mTOR pathway inhibitors, as well as lysosomal inhibitors. These are usually combined with other anti-cancer therapies, such as chemotherapeutic drugs, immunotherapeutic agents, or endocrine therapies. For example, the AMPK activator metformin has shown potential for combined application with chemotherapy or immunotherapy in clinical trials of breast cancer, colorectal cancer, and head and neck cancer; while mTOR inhibitors such as everolimus and rapamycin have been widely tested in clinical trials of multiple cancers, including breast cancer, colorectal cancer, liver cancer, and prostate cancer. These research findings suggest that autophagy-targeting drugs have broad application prospects in the treatment of various cancers. However, clinical trial results also indicate that the efficacy of autophagy inhibitors may be affected by tumor type, disease stage, and individual patient differences. Therefore, strategies targeting autophagy require precise design based on specific cancer types and therapeutic contexts.

**Table 4:** Clinical trials of cancer therapy targeting autophagy.

Target	Drug targeting autophagy	Cancer type	Combination therapy	Phase	Trial ID
AMPK	Metformin	Breast Cancer	Erlotinib/ None/ None/ Chemotherapy	I/ II/ III/ IV	NCT01650506/ NCT01266486/ NCT01101438/ NCT05840068
		Colorectal Cancer	Nivolumab/ None	II/ III	NCT03800602/ NCT05921942
		Head and Neck Cancer	Cisplatin, Radiation/ None	I/ I, II	NCT02325401/ NCT02949700
		Prostate Cancer	Docetaxel/ None	II/ II	NCT01796028/ NCT01243385
	Resveratrol (SRT501)	Colon Cancer	None	I	NCT00256334
		Colorectal Cancer	None/ None	I/ I	NCT00433576/ NCT00920803
		Multiple Myeloma	Bortezomib	II	NCT00920556
mTOR	Everolimus (RAD001)	Breast Cancer	Cisplatin, Paclitaxel/ Fulvestrant/ Exemestane/ None	I/ II/ III/ IV	NCT00680758/ NCT00570921/ NCT00863655/ NCT01948960
		Colorectal Cancer	Bevacizumab/ None	II/ II	NCT00597506/ NCT00419159
		Liver Cancer	sorafenib tosylate/letrozole, leuprolide/ None	II/ II/ I, II	NCT01005199/ NCT01642186/ NCT00390195
		Prostate Cancer	Enzalutamide/ Bicalutamide	I/ II	NCT02125084/ NCT00814788
		Renal Cell Carcinoma	None/ None/ None/ None	I/ I, II/ III/ IV	NCT01442090/ NCT01462214/ NCT01865747/ NCT02056587
	Rapamycin (Sirolimus)	Acute Lymphoblastic Leukemia	Corticosteroid/ None	I/ II	NCT00874562/ NCT00795886
		Acute Myeloid Leukemia	Decitabine/ None	I/ II	NCT00861874/ NCT00235560
		Breast Cancer	Trastuzumab/ Inetetamab	II/ III	NCT00411788/ NCT04736589
		Liver Cancer	Bevacizumab	I	NCT00467194
		Lung Cancer	Sunitinib/ BIBW 2992	I/ I	NCT00555256/ NCT00993499
		Prostate Cancer	None	I, II/ I	NCT00311623/ NCT03618355
	Ridaforolimus (MK-8669)	Breast Cancer	Dalotuzumab/ Trastuzumab	I/ II	NCT01220570/ NCT00736970
		Endometrial Cancer	Carboplatin, Paclitaxel/ None	I/ II	NCT01256268/ NCT00122343
		Prostate Cancer	Bicalutamide/ None	II/ II	NCT00777959/ NCT00110188
PI3K/mTOR	Apitolisib (GDC-0980)	Breast Cancer	Fulvestrant/ Paclitaxel, Bevacizumab	II/ I	NCT01437566/ NCT01254526
		Endometrial Carcinoma	None	II	NCT01455493
		Non-Hodgkin’s Lymphoma, Solid Cancers	None/ None	I/ I	NCT00854126/ NCT00854152
		Prostate Cancer	Abiraterone Acetate	I/ II	NCT01485861
		Renal Cell Carcinoma	None	I	NCT01442090
	BGT226	Advanced Solid Tumor	None	I	NCT00742105
		Breast Cancer	None	I, II	NCT00600275
	Dactolisib (BEZ235)	Advanced Solid Tumor	None	I	NCT01195376
		Breast Cancer	None/ Letrozole	I, II/ I	NCT00620594/ NCT01248494
		Prostate Cancer	Abiraterone Acetate	I	NCT01634061
	PF-04691502	Advanced Solid Tumor	None	I	NCT00927823
	PF-05212384 (Gedatolisib)	Breast Cancer	Palbociclib, Letrozole, Fulvestrant/ Fulvestrant, Palbociclib	I/ III	NCT02684032/NCT05501886
		Solid Tumor	None/ Paclitaxel, Carboplatin	I/ I	NCT00940498/ NCT02069158
Lysosomal	Chloroquine	Breast Cancer	Taxane-Like Chemotherapy	II	NCT01446016
		Ductal Carcinoma In Situ	None	I, II	NCT01023477
		Glioblastoma Multiforme	Radiotherapy, Temozolomide	I, III	NCT02378532/ NCT00224978
		Pancreatic Cancer	Gemcitabine	I	NCT01777477
		Solid Tumors	Carboplatin, Gemcitabine	I	NCT02071537
	Hydroxychloroquine	Breast cancer	Palbociclib/ Letrozole, Palbociclib	I, II/ I, II	NCT05953350/ NCT03774472
		Cholangiocarcinoma	Trametinib/ ABC294640	II/ II	NCT04566133/ NCT03377179
		Colorectal Cancer	Capecitabine, Oxaliplatin, Bevacizumab/ Vorinostat	II/ II	NCT01006369/ NCT02316340
		Gastrointestinal Cancer	Cobimetinib, Atezolizumab/ Ulixertinib	I, II/ II	NCT04214418/ NCT05221320
		Glioblastoma	Radiation, Temozolomide	I, II	NCT00486603
		Hepatocellular Cancer	Lipiodol/ Sorafenib	I, II/ II	NCT05842174/ NCT03037437
		Lung Cancer	Carboplatin, Paclitaxel, Bevacizumab/ Carboplatin, Paclitaxel, Bevacizumab	II/ I, II	NCT01649947/ NCT00728845
		Melanoma	None/ Dabrafenib, Trametinib/ Dabrafenib, Trametinib	I/ I, II/ II	NCT00962845/ NCT03754179/ NCT04527549
		Pancreatic Cancer	Binimetinib/ Gemcitabine, Abraxane/ Capecitabine, Radiation	I/ I, II/ II	NCT04132505/ NCT01506973/ NCT01494155
		Prostate Cancer	None/ Docetaxel	I/ II	NCT02421575/ NCT00786682
		Renal Cell Carcinoma	None	I	NCT01144169

### Targeting acetylation–autophagy crosstalk in cancer therapy

**Fig. 5:**
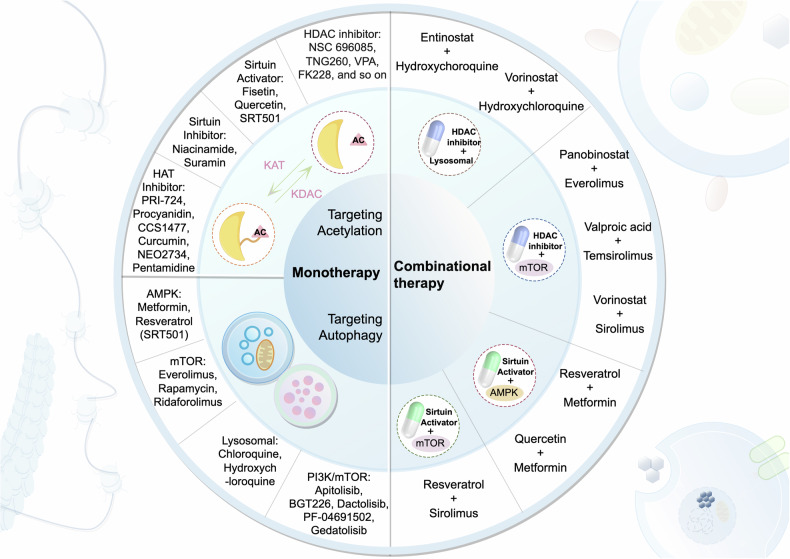
Cancer therapy targeting acetylation and autophagy.

The regulation of acetylation and autophagy, two critical cellular biological processes, holds significant importance in cancer therapy. An increasing number of studies have shown that drugs jointly targeting acetylation and autophagy have the potential to enhance anti-cancer activity, particularly across multiple cancer types. In clinical therapy, targeting acetylation and autophagy mainly involves monotherapy and combination therapy (Fig. 5). Monotherapy primarily focuses on activators and inhibitors targeting different families of acetyltransferases and deacetylases, as well as specific activators/inhibitors designed for different signaling pathways involved in the autophagy process. In combination therapy, current clinical trials predominantly follow four major strategies: the combination of HDAC inhibitors with autophagy modulators targeting lysosomes; HDAC inhibitors combined with autophagy modulators targeting the mTOR signaling pathway; SIRT family activators combined with autophagy modulators targeting the AMPK signaling pathway; and SIRT family activators combined with autophagy modulators targeting the mTOR signaling pathway.

Data in Table 5 indicate that HDAC inhibitors such as SAHA (Vorinostat) and Panobinostat, when used together with autophagy modulators like chloroquine, can significantly enhance apoptosis and autophagic responses in cancer cells, thereby inhibiting tumor growth [113, 114]. Specifically, the combined treatment of SAHA and chloroquine has been shown to increase autophagic volume in cancer cells, suppress tumor proliferation, and simultaneously promote tumor cell apoptosis [115]. In addition, the combination of SAHA and Everolimus has demonstrated enhanced autophagy in various tumor cell lines, contributing to improved efficacy of anti-cancer drugs [116]. Another class of drugs, such as SRT1720 and CF-Eos, has also shown the ability to suppress tumor growth by jointly targeting autophagy and acetylation pathways. SRT1720, through activation of SIRT1, regulates the expression of autophagy-related proteins and alters acetylation modifications during autophagy, thereby affecting autophagic flux. This mechanism plays an important role in combating bladder cancer and other tumor types [117–119]. Furthermore, other combination therapies, such as Resveratrol, Valproic acid, and Temsirolimus, have also demonstrated promising anti-cancer effects. Resveratrol enhances autophagy activity and anti-tumor effects by increasing autophagosome accumulation and modulating the acetylation of SIRT1 and other autophagy-related proteins [38, 120, 121]. The combination of Valproic acid and Temsirolimus effectively elevates autophagy levels in solid tumors and overcomes drug resistance [122, 123]. These research findings suggest that strategies jointly targeting acetylation and autophagy are not only validated in basic research but also hold clinical application potential.

**Table 5:** Clinical trials of cancer therapy targeting the acetylation-autophagy axis.

Drug targeting acetylation	Drug targeting autophagy	Status	Cancer Type	Trial ID	Phase
Entinostat	Hydroxychloroquine	Completed	Colorectal Cancer	NCT03215264	I
Panobinostat	Everolimus	Completed	Advanced Solid Tumors	NCT01055795	I
Panobinostat	Everolimus	Terminated	Diffuse Large B-Cell Lymphoma	NCT00978432	II
Panobinostat	Everolimus	Completed	Lymphoma	NCT00967044	I/II
Panobinostat	Everolimus	Completed	Lymphoma	NCT00962507	I
Panobinostat	Everolimus	Completed	Relapsed Multiple Myeloma or Lymphoma	NCT00918333	I/II
Panobinostat	Everolimus	Terminated	Renal Cell Carcinoma	NCT01582009	I/II
Panobinostat	Everolimus	Unknown status	Solid Tumors/ Lymphomas	NCT01341834	I
Quercetin	Metformin	Unknown status	Breast Cancer	NCT05680662	I
Resveratrol	Metformin	Completed	Lymphangioleiomyomatosis	NCT04867252	II
Resveratrol	Sirolimus	Completed	Lymphangioleiomyomatosis	NCT03253913	II
Resveratrol	Sirolimus	Completed	Lymphangioleiomyomatosis	NCT03253913	II
Valproic acid	Temsirolimus	Active, not recruiting	Advanced or Metastatic Malignancy	NCT01552434	I
Valproic acid	Temsirolimus	Terminated	Multiply Relapsed Pediatric Solid Tumors	NCT01204450	I
Valproic acid	Temsirolimus	Recruiting	Solid Tumor	NCT02446431	I
Vorinostat	Hydroxychloroquine	Completed	Colorectal Cancer	NCT02316340	II
Vorinostat	Hydroxychloroquine	Completed	Malignant Solid Tumor	NCT01023737	I
Vorinostat	Hydroxychloroquine/Sirolimus	Completed	Advanced Cancers	NCT01266057	I
Vorinostat	Ridaforolimus	Completed	Advanced Solid Tumors or Lymphoma	NCT01169532	I
Vorinostat	Sirolimus/Everolimus/Temsirolimus	Active, not recruiting	Advanced Cancer	NCT01087554	I
Vorinostat	Temsirolimus	Active, not recruiting	Diffuse Intrinsic Pontine Glioma	NCT02420613	I
Vorinostat	Temsirolimus	Terminated	Metastatic Prostate Cancer	NCT01174199	I

## Conclusion and prospects

The crosstalk between acetylation modification and autophagy constitutes a core mechanism in the regulation of cellular homeostasis. This review systematically summarizes their dynamic balance under physiological and pathological conditions, as well as their complex biological functions in cancer initiation, progression, drug resistance, prognosis, and tumor microenvironment regulation. Under physiological conditions, acetylation modification directly influences the initiation and termination of autophagy by modulating the activity and stability of autophagy-related proteins, while autophagy indirectly maintains the dynamic balance of acetylation through degradation of acetyltransferases/deacetylases or regulation of metabolic substrates. In cancer, this bidirectional regulatory network significantly impacts tumor cell survival, metastasis, and drug resistance through mechanisms such as metabolic reprogramming, DNA repair, and immune microenvironment remodeling. Currently, combined therapeutic strategies targeting acetylation and autophagy have shown significant anti-cancer potential in both preclinical studies and clinical trials.

Despite the important breakthroughs achieved in elucidating the crosstalk mechanisms between acetylation and autophagy, multiple bottlenecks continue to limit both the depth of basic research and clinical translational potential. [1] Insufficient depth and breadth of mechanistic research. Current work has mainly centered on the acetylation of core autophagy proteins in mammalian cells, with little effort to systematically assess the conservation or divergence of this pathway in plants or lower organisms. Such bias limits our understanding of whether the acetylation–autophagy axis represents a universal regulatory principle or a lineage-specific adaptation. Moreover, most studies focus narrowly on lysine acetylation, whereas irreversible modifications such as N-terminal acetylation—likely to affect protein stability and localization—remain poorly characterized in the context of autophagy [124]. These gaps highlight the need for cross-species comparative genomics to clarify evolutionary conservation, mechanistic dissection of non-classical acetylation in autophagosome biogenesis and substrate selection, and exploration of whether acetylation–autophagy crosstalk operates differently in organelle-specific autophagy. [2] Technical limitations constrain mechanistic insights. The dynamic and spatial specificity of acetylation modifications impose high demands on detection technologies. Although current mass spectrometry techniques allow high-throughput identification of acetylation sites, they struggle to capture transient modifications or dynamic acetylation events in micro-compartments [125]. Additionally, the heterogeneity of autophagy means traditional bulk-cell analyses may obscure key regulatory details. Combining single-cell sequencing with spatial omics could reveal cell-type-specific regulatory patterns of the acetylation-autophagy axis within the tumor microenvironment. Future efforts should focus on developing high spatiotemporal resolution imaging technologies for dynamic acetylation (e.g., live-cell tracking using fluorescent probes); integrating single-cell multi-omics (epigenomics, metabolomics) to analyze heterogeneity of the acetylation-autophagy axis in the tumor microenvironment; and employing organoid and 3D culture models to simulate in vivo environments for studying the mechanical and biochemical regulation of acetylation-autophagy interactions [126]. [3] Challenges in drug development and clinical translation. Although drugs targeting acetylation or autophagy-lysosome pathways have entered clinical trials, their efficacy is often limited by off-target effects and tumor adaptive resistance. Importantly, the acetylation–autophagy crosstalk itself complicates therapeutic targeting: global HDAC inhibition not only alters histone acetylation but can also reprogram autophagy toward pro-survival functions, while autophagy inhibition may produce opposite outcomes depending on tumor type and disease stage. These challenges highlight the need for strategies that specifically exploit acetylation–autophagy crosstalk, such as developing subtype-selective enzyme modulators, designing spatiotemporally controlled delivery systems, and combining acetylation regulators with autophagy modulators or metabolic inhibitors to achieve synthetic lethality and improve efficacy. [4] Complexity of tumor microenvironment and immune regulation. The acetylation-autophagy axis not only regulates tumor cells but also affects immune cell function. This interplay influences tumor invasion, metastasis, and immune evasion via modulation of metabolic pathways, signaling networks, and epigenetic states. In immune cells, autophagy coupled with histone deacetylation or acetyltransferase modulation can alter CD8⁺ T cell cytotoxicity and stemness, Treg suppression, antigen presentation, and macrophage activation [87–94, 127]. However, the precise mechanisms by which acetylation–autophagy interactions control immune checkpoint stability and orchestrate anti-tumor immunity remain unclear. Future studies should elucidate these mechanisms in diverse immune subsets and explore combination therapies that target the acetylation–autophagy axis to reverse immunosuppression and enhance immunotherapy efficacy. [5] Precision and personalization in clinical translation. The clinical translation of acetylation–autophagy crosstalk remains limited by tumor heterogeneity and the lack of robust, standardized biomarkers. Future strategies should focus on developing multi-modal models integrating acetylome profiling, autophagy flux markers, and functional readouts to predict therapeutic responses dynamically. Liquid biopsy–based combinational biomarkers, such as exosomal acetylated RNAs coupled with autophagy-related proteins, can enable non-invasive monitoring and guide individualized therapy. Patient-derived xenograft or organoid models may facilitate preclinical screening of personalized combination regimens targeting the acetylation–autophagy axis. Integrating computational modeling and experimental oncology will be critical for establishing precision treatment decision-making systems based on this crosstalk.

In conclusion, research on the interaction mechanisms between acetylation and autophagy is at a critical stage of transitioning from basic science to clinical translation. Future progress requires technological innovation and interdisciplinary integration to overcome bottlenecks in mechanistic elucidation and drug development, ultimately achieving precision cancer therapy based on the crosstalk between acetylation and autophagy.

## Data Availability

Data sharing not applicable to this article as no datasets were generated or analyzed during the current study.
